# Adhesion and Energy Characteristics of Rigid-Chain Polymer Surface: Polyamidoimides

**DOI:** 10.3390/polym12122956

**Published:** 2020-12-10

**Authors:** Anatoly E. Chalykh, Valentina Yu. Stepanenko, Ali D. Aliev

**Affiliations:** Frumkin Institute of Physical Chemistry and Electrochemistry of Russian Academy of Sciences, Leninsky pr. 31-4, 119071 Moscow, Russia; 4niko7@list.ru (V.Y.S.); ali_aliev1948@mail.ru (A.D.A.)

**Keywords:** polyamidoimide, surface energy, polar and dispersion components, adhesion

## Abstract

The adhesion characteristics and surface energies of two series of polyamidoimides (PAI) with different molecular weights, monomer unit structures, hinge groups in the main chain of the macromolecules, and thermal prehistory were determined via delamination at 180° and test fluids contact angles. We found that PAI are high-energy polymers, the surface energy of which varies in the range from 32 to 45 mJ/m^2^. In contrast to flexible-chain polymers, the exponent in the McLeod equation is two, which is due to the flat parallel orientation of the macromolecular chains in the surface layers. The main contribution to the change in surface characteristics of these polymers is the change in the packing density of PAI macromolecules, which is reflected mainly in the change in the polymers’ dispersion component. We found that the adhesion properties of PAI with respect to high- and low-energy substrates are determined mainly by the macromolecules packing density in the surface layers with their conformation state unchanged.

## 1. Introduction

Polyamidoimides (PAI) are high-molecular compounds, the monomer units of which consist of aromatic carbo- and heterocycles [1,2]. They are characterized by high thermal, radiation, and chemical resistance; they can form films, fibers, membranes, and coatings with uniquely high-strength, protective, and gas separation properties. Their application as high-temperature adhesives, binding engineering plastics, electrically conductive materials, protective coatings, and elements of optical devices has been described in the literature [3,4,5,6]. Their application in materials and elements of aerospace structures has also been described [1,7,8].

PAI belong to thermodynamically rigid-chain polymers with a Kuhn segment of 18–20 nm [9]. Like other rigid-chain polymers, they include flat cyclic groups that are connected with each other by bridge atoms and groups that largely determine the operational properties of polymers. The glass transition temperature, which characterizes the kinetic mobility of segments depending on the composition and structure of radicals in the structure of the monomer units, varies in the range of 490–590 K [1,2,9]. A unique feature of PAI is that there is a rather high local mobility of fragments of macromolecular chains, which is associated with transitions of trans-gauche conformers due to hinge vibrations of bridge atoms near the amide and carbonyl groups. It was established [2,9] that low-temperature γ-transition associated with the rotations of aryl radicals in the C_Ar_–C_Ar_, C_Ar_–CO–C_Ar_, and C_Ar_–O–C_Ar_ structures is characterized by activation energy of 40–60 kJ/mol; the β-transition observed in the region of 290–320 K is characterized by ca. 140 kJ/mol and α by ca. 550 kJ/mol. Thus, the thermal mobility unfreezing process consequently involves structural elements of different scales: para-phenylene cores of diamine and phthalimide cycles of anhydride fragments, and cooperative motions of macromolecular chain segments as a result of dissociation of hydrogen bond grid.

Structural-morphological and diffraction studies have shown [10,11,12] that PAI crystalline regions consist of strictly ordered parallel phenyl and heterochain groups, while amorphous regions, the size of which is comparable to the size of 2–3 monomer units, are characterized by parallel stacking of straightened chains. While the physical-mechanical, relaxation, and sorption properties of PAI have been studied and described in sufficient detail, the information on surface and adhesion characteristics of polyesterimides that determine the possibility and prospects of their use as adhesives is quite fragmentary [3,7].

The present work provides data on the surface energy characteristics of PAI of different molecular weights, structures of monomer unit, and strength of adhesion to high- and low-energy substrates. Such studies are needed because there is no consensus about the absence of correlation between the energy characteristics of the adhesive and the strength of adhesive joints. However, such a correlation has been established in previous studies [13,14].

## 2. Experimental

**Figure 1:**
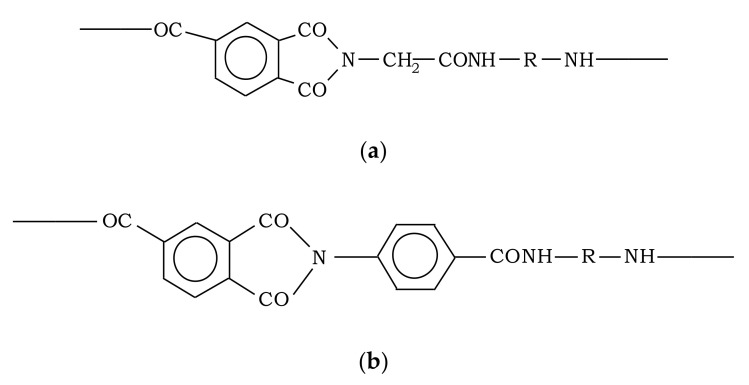

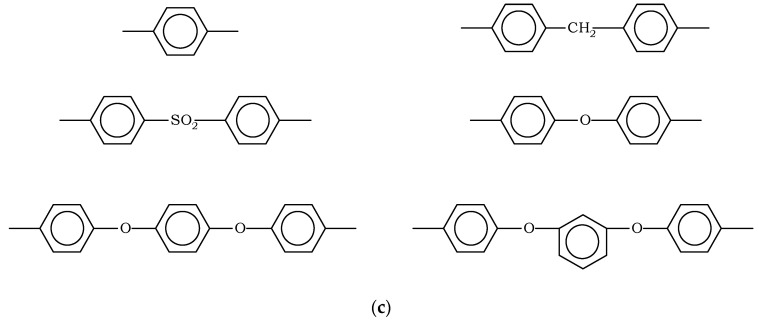
Chemical structure of monomer units of polyamidoimides: (**a**) PAI-A, (**b**) PAI-B, and (**c**) R—aromatic radical of the diamine fragment.

Polyamidoimides (Institute of High-Molecular Compounds Chemistry of National Academy of Science of Ukraine, Kiev), synthesized by low-temperature polycondensation of dichloroanhydrides of acetic (PAI-A, Figure 1a) and benzoic (PAI-B, Figure 1b) acids with aromatic diamines (Figure 1c) in solution of *n*-methylpyrrolidone, were used as objects of this study. The polymers were fractionated by three-fold transfer from *n*-methylpyrrolidone to ethyl alcohol. The characteristics of the investigated samples are given in Table 1.

PAI films with thickness of 50–80 µm were obtained by applying a 15% polymer solution in dimethylformamide onto glass and PET (polyethylene terephthalate) substrates and further drying in a vacuum at ~370 K for 36 h. Then, the films were separated from the substrates and subjected to additional heat treatment at 523 K for 3 h. According to mass thermal analysis data, residual solvent content did not exceed 1–2% for this method of sample preparation. Control experiments by means of thermogravimetry method (Netzsch TG209F1 Iris, Selb, Germany) showed that evaporation of residual solvent captured by PAI macromolecules during the preparation of films and adhesives mainly occurs in the range of 400–490 K. Heat treatment at 523 K for three hours resulted in the increase in heat-treated series density by 1–7% on average.

The adhesive joints of PAI with high-energy substrates (glass, Al foils, and steel) and low-energy substrates (flexible PET and PTFE (polytetrafluoroethylene) substrates with thickness of 100 µm) were also obtained by applying 15% polymer solution in dimethylformamide with subsequent vacuum drying at ~370 K for 36 h and additional heat treatment at temperatures of 393, 473, and 523 K for 6 h. We assumed that the states of PAI in both cases were identical, which was confirmed by the closeness of the sample density. Thus, in the process of thermal annealing, we successively involved local and cooperative elements of PAI macromolecules of different scale into structural rearrangements.

For measurements of polymer surface energy and its polar and dispersion components, the method of contact angle measurements for PAI surface wetting by a set of test liquids with known characteristics (γ, γ_D_, γ_P_) was employed (Table 2) [14]. A drop of liquid with volume from 1 × 10^−3^ to 1 × 10^−2^ cm^3^ was applied to the investigated surface of polymer sample with a microsyringe. The contact angle values were determined using Easy Drop (KRUSS, Hamburg, Germany) device. All measurements were recorded at room temperature.

The data obtained by the contact angle method were processed using Owens and Wendt equation [14,15].

(1) $$1+\cos\theta =\frac{2{\left(\gamma_{D}^{s}\right)}^{1/2}{\left(\gamma_{D}^{lv}\right)}^{1/2}}{\gamma^{lv}}+\frac{2{\left(\gamma_{P}^{s}\right)}^{1/2}{\left(\gamma_{P}^{lv}\right)}^{1/2}}{\gamma^{lv}}$$

where $\gamma_{D}^{s}$, $\gamma_{P}^{s}$, $\gamma_{D}^{lv}$, and $\gamma_{P}^{lv}$ are the dispersion and polar components of the free surface energies of the solid and liquid phases, respectively.

**Figure 2:**
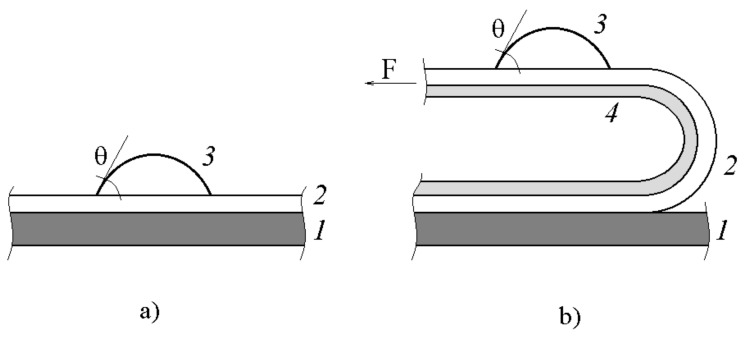
Adhesion joint testing. (**a**) Determination of contact angles of PAI adhesive films; (**b**) peeling of PAI adhesive reinforced by 3M tape. (1) Substrate, (2) PAI adhesive; (3) test liquid droplet; (4) 3M tape; F is peeling force applied at 180°.

Adhesion joints were tested by peeling PAI from substrates using 3M tape (Figure 2).

The peeling force was measured at room temperature using an Instron 1130 bursting machine (Instron, Norwood, MA, US) at a clamp travel speed of 10 mm/min. Samples with a lapping length of 5 cm, contact area of 6 cm^2^, and thickness of adhesive layer of 80–90 µm were used.

The integral density of the samples (ρ) was determined by the hydrostatic weighing and gradient column methods. The PAI surface and the nature of adhesive joint destruction were analyzed by X-ray photoelectron spectroscopy (XPS) and electron-probe X-ray microanalysis (EPMA) methods. Measurements were recorded using an XSAM-800 Kratos (Manchester, UK) in a vacuum of about 10^−10^ Pa. The photoelectron spectra were excited by characteristic radiation of magnesium (K Mg = 1253.6 mA). The power of the X-ray gun did not exceed 75 W. Charging of samples during registration of spectra was accounted for by carbon component with binding energy of 285.0 eV. Standard software package DS-800 (Manchester, UK) was used for photoelectronic spectra processing.

Braking X-ray radiation arising from the interaction of the electron beam with the oligomers surface was used to determine the density of the PAI surface layer (ρ_sur_). Measurements were performed using a scanning electron microscope equipped with an Eumex energy dispersive microanalyzer (Hamburg, Germany), at various accelerating voltages in the range of 9–25 keV. The method of sample preparation did not differ from the one described in [16]. Standard WINEDX software (Hamburg, Germany) was used for treatment of braking radiation spectra. Quantitative analysis was performed using the previously described technique [16], using radiation intensity–density calibration curves for different polymer materials. Error of the used method was ρ ± 0.01 g/cm^3^. Measurements were recorded in two modes: from a 5 × 5 µm area of and at a point (local area), the size of which corresponded to the diameter of the X-ray radiation generation zone. In the latter case, measurements were recorded for 20–30 randomly selected points on the sample surface.

## 3. Results and Discussion

**Figure 3:**
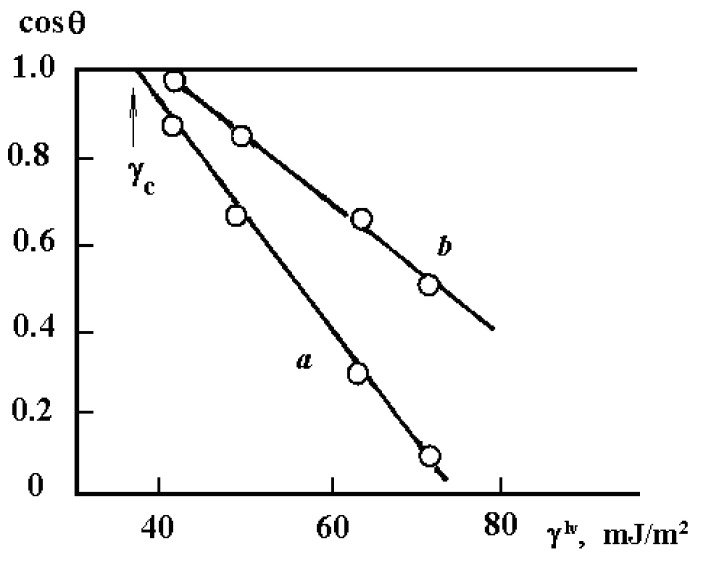
Dependence of cos θ on surface tension of liquids. (**a**) PAI-A and (**b**) PAI-B.

Table 3 shows the obtained values of surface energy, its polar and dispersion components for PAI different in structure, and composition of monomer units. For comparison, Table 4 presents data on critical surface tension determined according to the Zisman technique (Figure 3).

PAI can be assigned to high-energy polymeric substrates such as polycaprolactam (γ = 41.4 mJ/m^2^), polyacrylonitrile γ = 60 mJ/m^2^), phenol-resorcinol adhesive (γ = 52 mJ/m^2^), aromatic polyamides, etc. [14].

Changes in the composition and structure of a diamine fragment of PAI macromolecule repeating unit by introducing flexible –СН_2_–, –О–, and –SO_2_– units affected the values of surface energy and polarity of polyamidoimides insignificantly.

A similar situation was observed earlier [13] for another rigid-chain polymer: polynaphthoylbenzimidazole and its copolymers.

The introduction of rigid *n*-benzamide units, rod-shaped fragments of biphenyl, into the chain affected the strength and deformation properties of the corresponding PAI, but the change in their surface energy and its polar component did not exceed 5–10%.

Only two chemical structures of monomer units, containing a sulfo-group in salt form –${\mathrm{SO}}_{3}^{-}\cdot {\mathrm{HNR}}_{3}^{+}$ and a –СH_2_– unit, were characterized by a significant excess of surface tension values in comparison to the average value. In the first case, this excess was due to the high polar component of the functional groups, and in the second, it was due to the packing density of PAI surface segments. XPS data confirmed the presence of the –${\;\mathrm{SO}}_{3}^{-}\cdot {\mathrm{HNR}}_{3}^{+}$ fragment in the surface layer (in the amount of 0.8–1.6% at.), and the X-ray structure analysis data for PAI indicated the presence of a more ordered mesomorphic phase in the volume and on the polymer surface [18,19].

**Figure 4:**
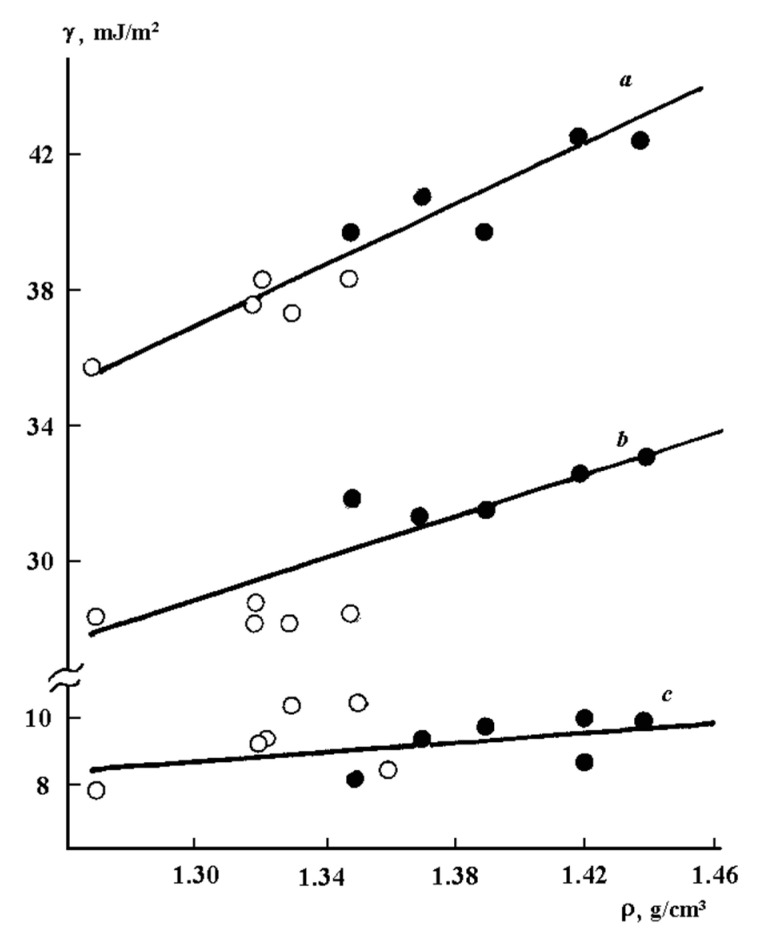
Dependence of (**a**) surface energy, (**b**) its dispersion, and (**c**) polar components on PAI density. ο—before annealing; ●—after annealing at 523 К, 3 h.

A fundamentally different effect on the surface properties of rigid-chain polymers was produced by their thermal annealing and the nature of the contact surface, the effect of which was assessed by the values of the energy characteristics of the surface of PAI adhesives after their delamination from the substrates (Figure 3). If thermal annealing of flexible-chain polymers, as a rule, led to changes in polar and dispersion components [3,13], in the case of PAI, thermal annealing of free films and adhesives, change of the contacting surface nature, and treatment of its surface with organic solvents of different polarity practically did not affect the surface energy polar component, while the surface energy dispersion component characterizing the chains packing density increased from 29 to 35 mJ/m^2^ (Figure 4) We assume that this effect is related to the invariability of the conformational set of macromolecule fragments in the PAI surface layer: the contour length of a segment of a persistent segment of a chain, the angle between the direction of a persistent segment at its beginning and at its end, the cosine value of the angle averaged throughout the chain, and the square of the distance between the ends of a worm-like chain.

The main contribution to the change in the surface characteristics of these polymers was the change in the packing density of PAI macromolecules, which was reflected mainly in the change in the dispersion component of the polymers. We can recall that the general structural kinetic model of the PAI structure suggests a slowdown of the torsional vibrations of phenylene rings, parallel positioning of phenylene and heterochain groups in distortedly parallel regions of amorphous polymers [18,19]. These results led us to assume that the surface activity of all groups that compose the monomer unit is constant and weakly depends on the thermal prehistory of the rigid-chain adhesive. These conclusions were confirmed by XPS data [20].

The second feature of behavior of rigid-chain polymers is their deviation from the McLeod law: $\gamma =\gamma_{0}\rho^{n}$, where $\gamma_{0}$ and *n* are positive temperature-independent constants. If, for the majority of flexible-chain polymers, we have *n* = 4 (Table 5), then we have *n* = 2 for the studied PAI. We attributed this result to the lower mobility of fragments of the macromolecular chains and, as a consequence, to the formation of extended oriented paracrystalline parquet-type structures.

Thus, it can be assumed that PAI segments and macromolecules as a whole, lacking the possibility for translational motion (at the interlayer distance of less than ca. 0.5 nm, the value of the barrier of inhibited internal rotation of macromolecule segments is 400 kcal/mol [21]), minimize their surface energy, probably due to self-organization of the paracrystalline packing of surface layers, thickness of which amounts to 1.5–2.0 nm, while maintaining an elongated conformation of the chains.

**Figure 5:**
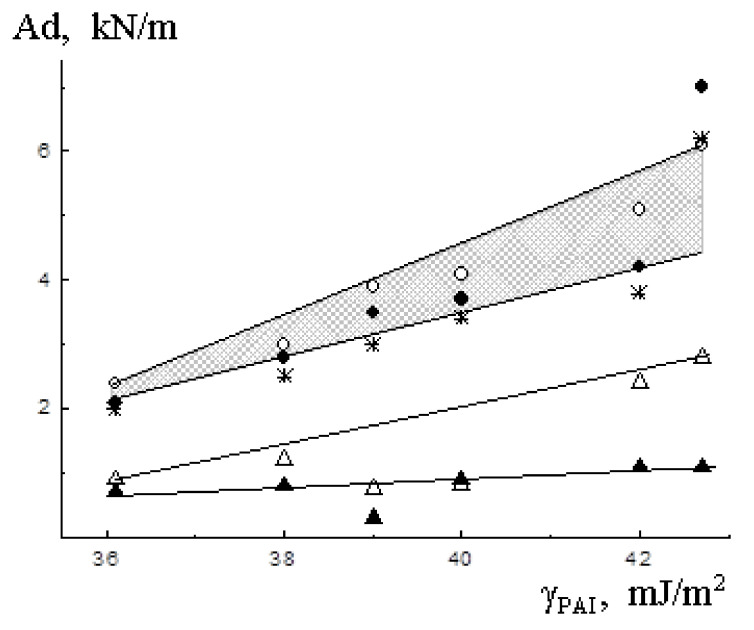
Dependence of the peeling force on the PAI surface energy for different substrates: (ο) aluminum, (●) steel, (*) glass, (Δ) PET, (▲) PTFE.

The dependence of the peeling force (σ) of PAI from high- (aluminum, steel, glass) and low-energy substrates (PET, PTFE) is expressed, as shown in Figure 5, by straight lines. For adhesive joints with high-energy substrates, the points on the dependence σ–γ plots formed narrow straight bands, within which all the test results were concentrated, i.e., the results obtained earlier are confirmed [13,14]. The slope angles of the σ–γ dependencies depend on the solubility parameter of the substrates, which agrees with the conclusions of Griffith-Irwin theory [13].

As we observed the destruction of the joints along the interface between the adhesive and the substrate for all adhesive systems, which we determined by the contact angle values for the substrate surfaces and the adhesives after destruction (Figure 2b), and the coincidence of the polar component of the fractal surface of the adhesive with the polar component of the initial PAI samples, we assumed that upon formation of the adhesive joints from PAI solution, the conformational set of the adhesive and its paracrystalline order in the adhesive joint correspond to the liquid-crystalline structure of the macromolecules associates in PAI solutions [22].

## 4. Conclusions

The results showed that the described structure of the PAI interfacial layers spontaneously forms in the adhesive joints and is associated with layering and parallel arrangement of phenylene and heterochain groups, which stabilize the most advantageous conformations of the chains when the largest number of polarized layers is formed. This, in turn, showed that this formation mechanism of the final structure should be realized for other rigid-chain adhesives. This will require further studies to complement modern research into the synthesis of heat-resistant rigid-chain polymers.

## Figures and Tables

**Table 1:** Characteristics of the studied polyamidoimides (PAI).

Sample	M_w_ * (kDa)	T_g_ ** (K)	ρ *** (g/cm^3^)
PAI-A1	24	565	1.397
PAI-A2	29	538	1.277
PAI-A3	30	505	1.329
PAI-A4	35	535	1.362
PAI-A5	38	520	1.342
PAI-A6	30	493	1.338
PAI-B1	23	593	1.379
PAI-B2	33	491	1.381
PAI-B3	33	525	1.394
PAI-B6	29	490	1.365

* Determined by GPC (Gel permeation chromatography). ** Determined by DSC method (Netzsch DSC 204 F1 Phoenix, Selb, Germany). *** Determined by hydrostatic weighing method.

**Table 2:** Surface energy of the test liquids (mJ/m^2^) at 25 °C.

Liquid	γ	γ_D_	γ_P_
Water	72.2	22.0	50.2
Glycerol	64.0	34.0	30.0
Dimethylformamide	37.3	32.4	4.9
Tricresyl phosphate	40.7	36.2	4.5
Polypropylene glycol (MM = 200)	43.5	28.2	15.3
Diiodomethane	50.8	48.5	2.3

**Table 3:** Surface energy (mJ/m^2^) of PAI with different structure of monomer units.

Monomer Unit	γ	γ_D_	γ_P_
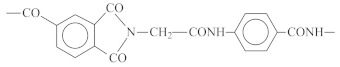	42.7/45.9 *	34.6/37.5	8.1/8.4
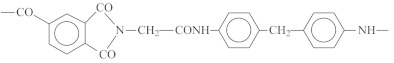	35.8/39.7	28.1/31.3	7.7/8.0
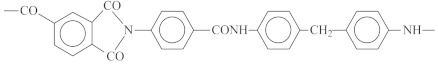	39.0/45.8	29./36.0	9.5/9.8
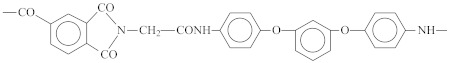	38.6/41.7	28.0/31.4	10.6/9.8
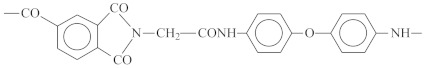	38.4/42.6	27.7/32.6	10.7/10.0
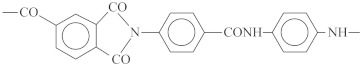	34.7/38.9	20.3/28.4	14.4/10.5
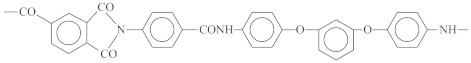	37.0/39.2	28.2/30.0	8.8/9.2
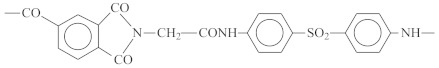	37.6/42.5	28.3/32.7	9.3/9.8
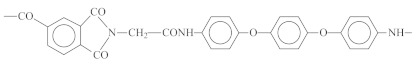	37.4/39.7	27.9/30.2	9.5/9.5
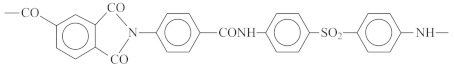	38.2/40.5	29.2/31.0	9.0/9.5

* before annealing/after annealing.

**Table 4:** PAI critical surface tension according by [17].

Sample	γ, mJ/m^2^	γ_c_, mJ/m^2^
PAI-A1	42.7	41.0
PAI-A3	38.6	38.0
PAI-A5	38.6	36.0
PAI-B1	34.7	33.0
PAI-B3	37.0	40.0

**Table 5:** Exponents in the McLeod equation.

Polymer	*n*	Reference
Polyamidoimide	2.3	our results
Poly(methyl methacrylate)	4.2	[13]
Poly-*n*-butylmethacrylate	4.2	[13]
Polystyrene	4.4	[13]
Polyvinyl acetate	3.4	[13]
Epoxide oligomer	3.8	[14,20]
